# Wall thickness analysis method for judging the degree of lower extremity long bone healing

**DOI:** 10.1038/s41598-023-48212-3

**Published:** 2023-11-24

**Authors:** Ying Li, Zhiwei Yang, Liangcheng Tong, Junsheng Yang, Jianling Wang, Yaoke Wen

**Affiliations:** 1 Department of Orthopedics, Air Force Hospital of Eastern Theater Command, Nanjing, Jiangsu China; 2 Department of Clinical Medicine, Anhui Medical University, Hefei, Anhui China; 3 School of Mechanical Engineering, Nanjing University of Science and Technology, No. 200 Xiaolingwei, Nanjing, 210094 Jiangsu China

**Keywords:** Medical research, Outcomes research

## Abstract

To evaluate the possibility of judging the degree of bone healing by wall thickness analysis provide reference for quantitative analysis of bone healing. Patients with lower limb fracture from April 2014 to October 2019 were recruited and divided into bone healing (group A), poor bone healing (group B), and nonunion (group C). Models were built in Mimics 20.0 with DICOM 3.0 data obtained from patient’s CT. Three-dimensional geometric models of unaffected limb and affected limb after simulated removal of internal fixation were established, corresponding to basic phase and simulated phase, respectively. Wall thickness analysis was performed to obtain median wall thickness after meshing. R2 (median wall thickness ratio), R4 (CT value ratio), and R5 (healing index ratio) were obtained by calculating the ratio of each value in simulated phase to that in basic phase. Receiver operating characteristic curve analysis was used to evaluate the ability of Wall Thickness Analysis to indicate fracture healing. 112 CT scans of 79 patients were included in the study. The frequency of categorization in groups A, B, and C was 49, 37 and 26, respectively. The median R2 in groups A, B, and C was 0.91, 0.80, and 0.67, respectively (group A > group B > group C, all *P* < 0.05). The best cutoff point for R2 in predicting bone healing was 0.84, and predicting bone nonunion was 0.74. The Wall Thickness Analysis can be used to quantitatively evaluate fracture healing state, with median wall thickness ratio as a more intuitive and reliable judgment index.

## Introduction

Approximately 6 million fractures occur in the United States each year, and 5–10% of patients eventually develop nonunion^1^. However, at present, there are no quantitative judgment standards for bone healing that can be recognized by orthopedic surgeons^2^. For the diagnosis of bone healing in fracture patients, most clinicians usually rely on clinical and imaging examinations, but the evaluation criteria vary greatly^3^. Therefore, Peter JO and Field JR started to use quantitative indicators to judge bone healing from the perspective of mechanics and imaging, respectively^4,5^. The fracture healing process is a continuous process over time^6^. Real-time quantitative observation of the degree of bone healing based on individualized imaging data is the direction of the development of methods for accurately studying the degree of bone healing.

The use of CT to observe the degree of bone healing after fracture is a commonly used technique in clinical practice. At present, CT is mainly used to observe the morphological changes in individual fracture areas^7^. In fact, CT provides information regarding the three-dimensional shape of the target bone segment, as well as instant density and thickness information. Shefelbine et al. used micro-CT and three-dimensional finite element analysis to evaluate the mechanical properties of the callus during fracture healing^8^. Andreas used finite element analysis to simulate the process of long bone callus formation and healing and believed that stress stimulation played an important role in the process of cell differentiation and fracture healing^9^. Some of the density and thickness information is lost in the finite element modeling process, and the simulation results are biased to a certain extent relative to the real results^10^. In fact, the original density and thickness information of the bone segment are included in the finite element differential modeling stage. At this stage, it is a simple and practical method to estimate the degree of bone healing in the injured limb by comparing the density and thickness of the injured limb with that of the uninjured side, and the periodic observation of individual wall thickness ratio changes over time helps to evaluate the degree of long bone healing in real time. We used the above methods to assist in evaluating the degree of bone healing in 79 clinical cases from 2014 to 2019, and the results are reported below.

## Methods

### Patients

The institutional review board of our hospital approved this study (Number 20150129). All patients provided written informed consent. From April 2014 to October 2019, patients with complete follow-up data who underwent internal fixation surgery for lower extremity fractures at our hospital were recruited. The follow-up data included X-ray and CT data 9 months after surgery. Two senior orthopedic doctors and an imaging physician judged the degree of fracture healing in the patients according to the patient’s X-ray, CT and clinical data. According to the above criteria, the status was judged as bone healing, poor bone healing and bone nonunion. If the diagnosis was inconsistent among the three doctors, the same result provided by two of the doctors was taken, and if the results of the three doctors were not the same, the case was excluded. A total of 79 patients were included in the study. A total of 112 CT scans were performed, 49 of the scans were judged to show bone healing (group A), 37 were judged to show poor bone healing (group B), and 26 were judged to show nonunion (group C). There were 21 females in group A, with an average age of 46.7 ± 12.4 years and a follow-up time of 18.7 ± 3.4 months. There were 12 cases of femoral fracture, 37 cases of tibial fracture, 15 cases of intramedullary nail fixation, and 34 cases of plate screw fixation in group A. There were 15 females in group B, with an average age of 51.2 ± 14.5 years and an average follow-up time of 14.7 ± 3.8 months. There were 9 cases of femoral fracture, 28 cases of tibial fracture, 24 cases of plate fixation, and 13 cases of intramedullary nail fixation in group B. There were 15 females in group C, with an average age of 49.2 ± 13.5 years and an average follow-up time of 15.7 ± 4.4 months. There were 8 cases of femoral fracture, 18 cases of tibial fracture, 17 cases of plate fixation, and 9 cases of intramedullary nail fixation in group C (Table 1). The fractures of the samples were diaphyseal and metaphyseal, not intra-articular fractures.

**Table 1:** Basic clinical data of patients.

Parameters	Group A (n = 49)	Group B (n = 37)	Group C (n = 26)
Age (years)	46.7 ± 12.4	51.2 ± 14.5	49.2 ± 13.5
Female sex (n, %)	21, 42.9%	15, 40.5%	15, 57.7%
Follow-up time (months)	18.7 ± 3.4	14.7 ± 3.8	15.7 ± 4.4
Location			
Femur (n, %)	12, 24.5%	9, 24.3%	8, 30.8%
Tibia (n, %)	37, 75.5%	28, 75.7%	18, 69.2%
Fixation method			
Plate (n, %)	34, 69.4%	24, 64.9%	17, 65.4%
Intramedullary nail (n, %)	15, 30.6%	13, 35.1%	9, 34.6%

### Hardware and software conditions

*Hardware*: GE 64-row spiral CT machine (Light Speed spiral CT, GE, USA). Dell high-performance computer (CPU: E3-1225 V2 3.20 GHz, memory: 16 GB, graphics card: NVIDIA Quadro K4200, operating system: Windows 10, 64-bit).

*Software*: Mimics Research 20.0, 3-matic Research 12.0 (Materialise, Belgium), provided by Sandi Tribe (Shanghai) Technology Co., Ltd.

### Data collection and processing

The lower limbs of the patients were placed in parallel with the toes up, and a full-length scan was performed. The CT scan interval was 0.625 mm, and the matrix size was 512 × 512 pixels. The scan voltage was 140 kV, the exposure was 100 mAs, and the screw pitch was 0.625 mm (GE 64-slice spiral CT machine with automatic tube current control system with the same scan parameters on both sides). The obtained general DICOM 3.0 standard format data were stored.

### Three-dimensional reconstruction

Mimics 20.0 software was used to directly read the CT images in DICOM 3.0 format. Three-dimensional geometric models were established under the same threshold conditions for the affected side with internal fixation, the affected side without internal fixation and the unaffected side, and the average CT data of the models were recorded.

### Model optimization

The three sets of data from the models of the healthy side, the affected side with internal fixation, and the affected side without internal fixation were imported into 3-matic Research 12.0. An adaptive triangle mesh was applied, and the side length was set to 1 mm to optimize the details of the model. The detailed characteristics of the model were retained, and the maximum wall thickness threshold was set to 10,000 mm during wall thickness analysis.

### Wall thickness analysis

To interpret the weak areas of the cortical bone in terms of the wall thickness and the transformation in terms of the relationship between the density and stiffness of the material, the median wall thickness of the unaffected limb and the affected limb with and without internal fixation was measured. The analysis was performed as follows: in the Analyze tab, the “Create Wall Thickness Analysis” button was clicked, and then “Cortical As Entity” was selected. The threshold was set to 10,000.0 mm. A histogram with the wall thickness distribution was displayed, and a series of colors was visualized on the Cortical 3D object, with green representing thinner structures and red corresponding to thicker areas.

**Figure 1:**
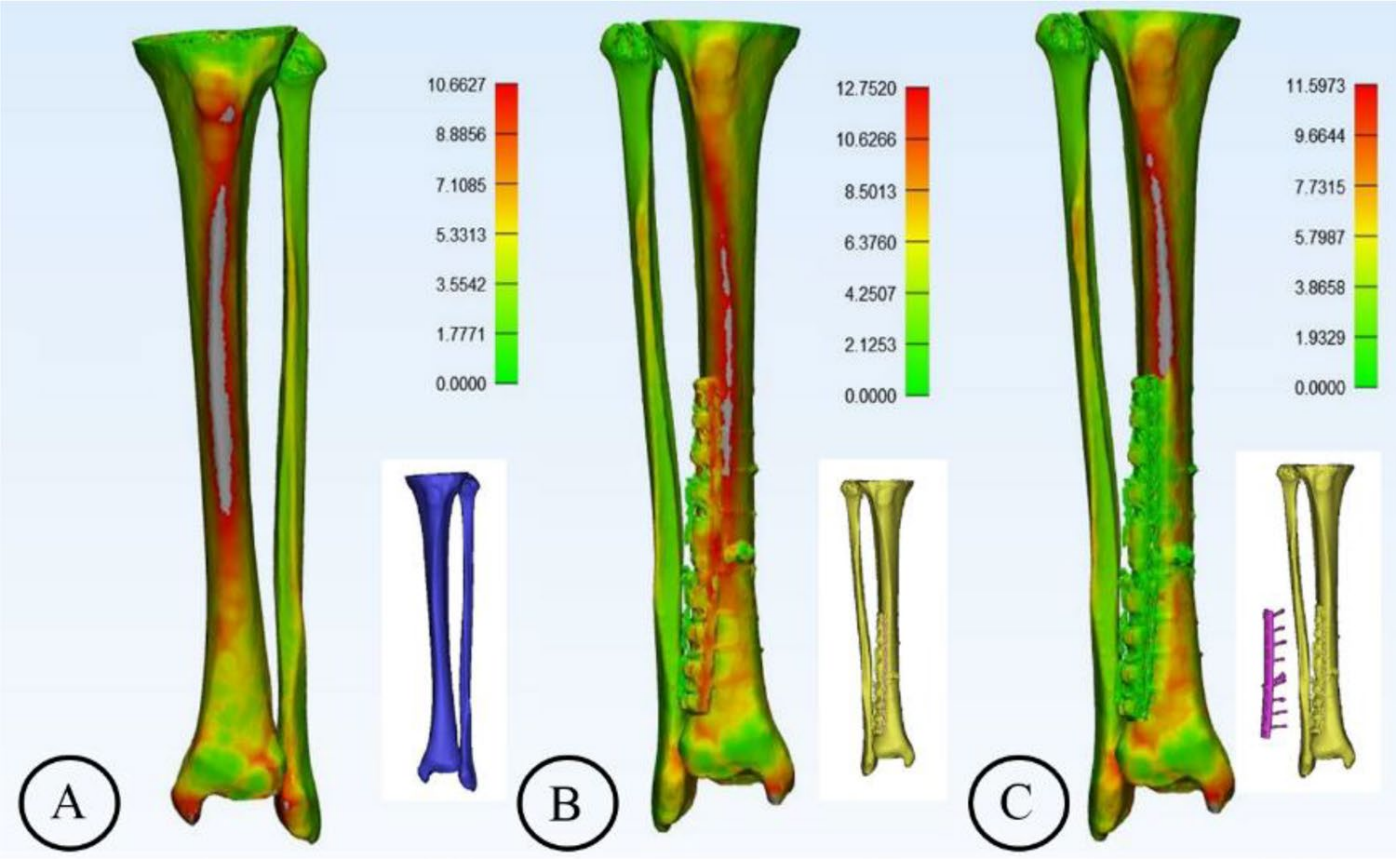
Three phases of wall thickness analysis: the basic phase (**A**), the target phase (**B**), and the simulated phase (**C**).

We performed three-dimensional reconstruction of the CT data of the healthy and affected segments under the same conditions, resulting in wall thickness graphs corresponding to the basic phase and the target phase, respectively. Three-dimensional reconstruction of the CT data of the affected limb with simulated removal of the internal fixation was performed, yielding a wall thickness graph corresponding to the simulated phase (Fig. 1).

### Observation index

The ratio of the median wall thickness and the average CT value in the simulated phase to the corresponding values in the basic phase was calculated to obtain the ratios R2 and R4: R2 = median wall thickness of the simulated phase/median wall thickness of the basic phase; R4 = average CT value of the simulated phase/average CT value of the basic phase. The product of the average CT value and the median wall thickness was defined as the healing index (HI), and R5 = simulated phase HI/basic phase HI.

### Follow-up method

The fracture healing state was evaluated through imaging and clinical examinations at 9 months after surgery. The bone healing of patients was observed for half a year after removal of the internal fixation to monitor for refracture. In the case that nonunion continued to be observed, bone grafting and internal fixation were performed again. Patients with poor bone healing were further observed. If there were no signs of healing, bone grafting was performed.

### Diagnostic criteria for bone healing and bone nonunion

The criteria for bone healing were as follows: X-ray images showed blurring of the fracture line and a continuous callus passing through the fracture line^11^; additionally, upon the release of external fixation, the patient does not have any tenderness at the fracture site, can walk with weight, and has no longitudinal pain in the fractured limb on percussion^12^.

The criteria for bone nonunion were as follows: Nonunion was defined by pain and abnormal activity at the fracture site, persistent light-transmitting bands on X-ray examination, and no progress in the formation of the callus at 12 weeks after treatment^13^.

Poor bone healing was defined as a healing state between that of bone healing and bone nonunion.

### Statistical analysis

Measurement data are expressed as the mean ± standard deviation or median, and Pearson or Spearman correlation analysis was performed. One-way ANOVA was conducted to analyze differences between multiple groups. Receiver operating characteristic (ROC) curve analysis was performed for the diagnostic analysis, and the critical point of diagnosis was analyzed by the maximum Youden index method. *P* < 0.05 was set as statistically significant, and all data analyses were performed by SPSS (version 20.0; IBM Corp., Armonk, NY, USA).

### Ethics approval and consent to participate

Informed consent was obtained from all the patients and the study was approved by Biomedical Ethics Committee of Anhui Medical University (reference number 20150129). The study has been performed in accordance with the ethical standards of the Declaration of Helsinki in 1964.

## Results

### Analysis of correlation and difference between degree of bone healing and observation index

**Figure 2:**
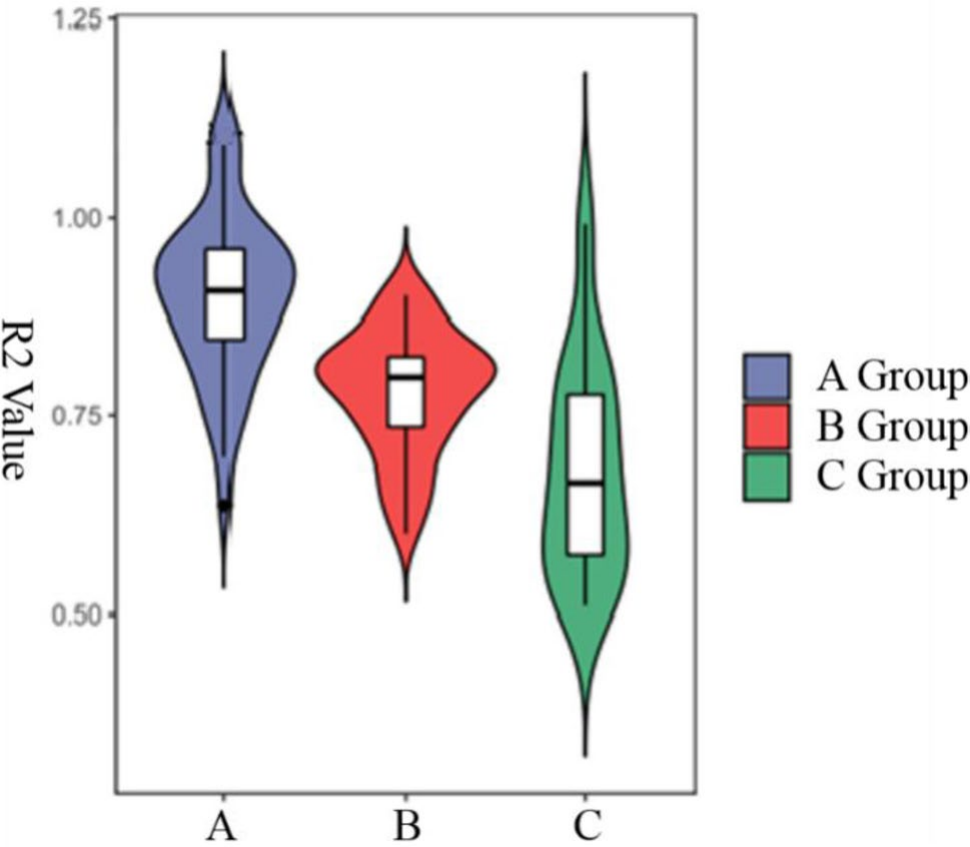
Comparison of R2 in groups A, B and C.

The Interclass Correlation Coefficient of the average CT value and the median wall thickness measured by the two doctors was 0.81 and 0.86, respectively, showing high repeatability. We carried out correlation analysis and one-way Interclass Correlation Coefficient of the degree of bone healing and R2, R4, and R5. R2, R4, and R5 did not follow a normal distribution. Correlation analysis showed a correlation coefficient of 0.060 between the degree of bone healing and R4 (*p* = 0.529), indicating no correlation. The correlation coefficient of the degree of healing with R2 and R5 was 0.654 (*P* < 0.001) and 0.542 (*P* < 0.001), respectively. The median R2 in groups A, B, and C was 0.907, 0.799, and 0.667, respectively, with a statistically significant difference among them (*p* < 0.05). Pairwise comparison showed that the median R2 in group A was significantly greater than that in groups B and C and that the median R2 in group B was significantly greater than that in group C (Fig. 2).

**Figure 3:**
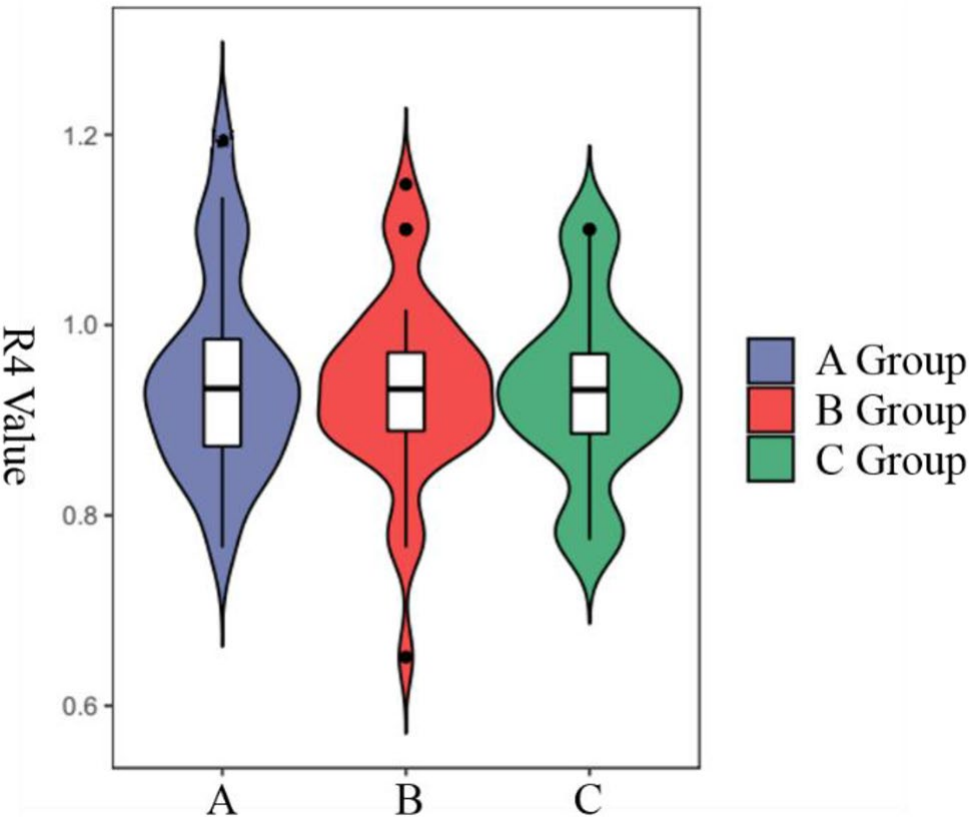
Comparison of R4 in groups A, B and C.

The median R4 in groups A, B, and C was 0.933, 0.932, and 0.932, respectively. There was no significant difference among the three groups (Fig. 3).

**Figure 4:**
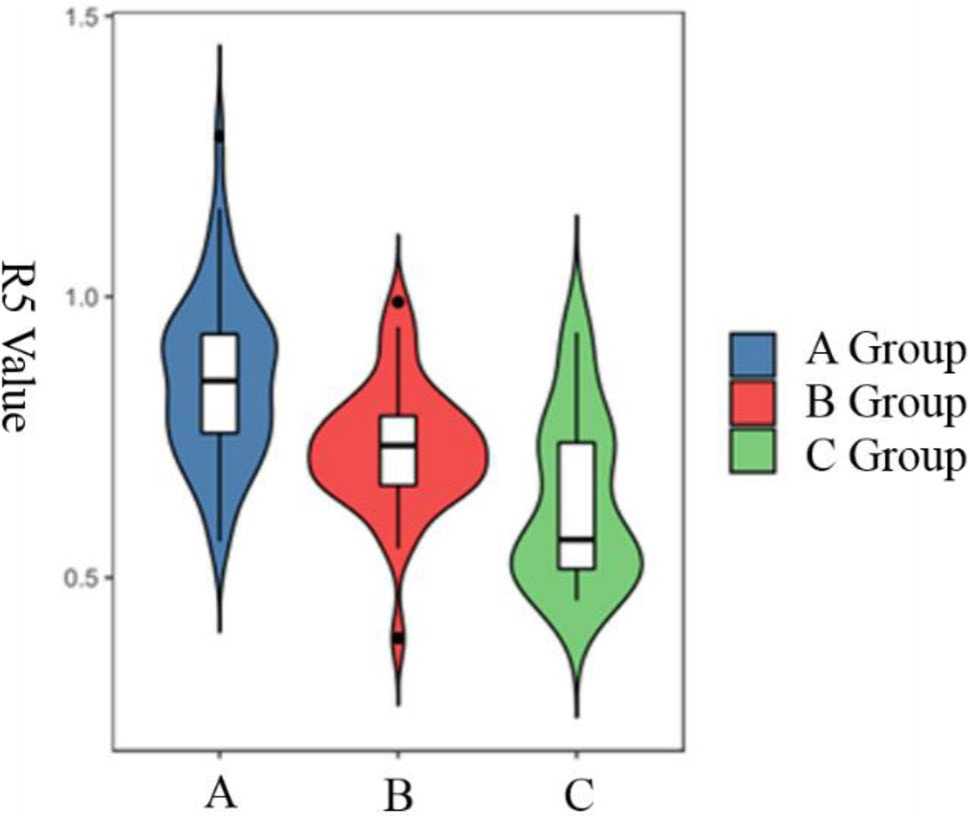
Comparison of R5 in groups A, B and C.

The median R5 in groups A, B, and C was 0.850, 0.735, and 0.568, respectively, with a statistically significant difference among them (*p* < 0.05). Pairwise comparison showed that the median R5 in group A was significantly greater than that in groups B and C and that the median R5 in group B was significantly greater than that in group C (Fig. 4).

### ROC curve analysis results

**Figure 5:**
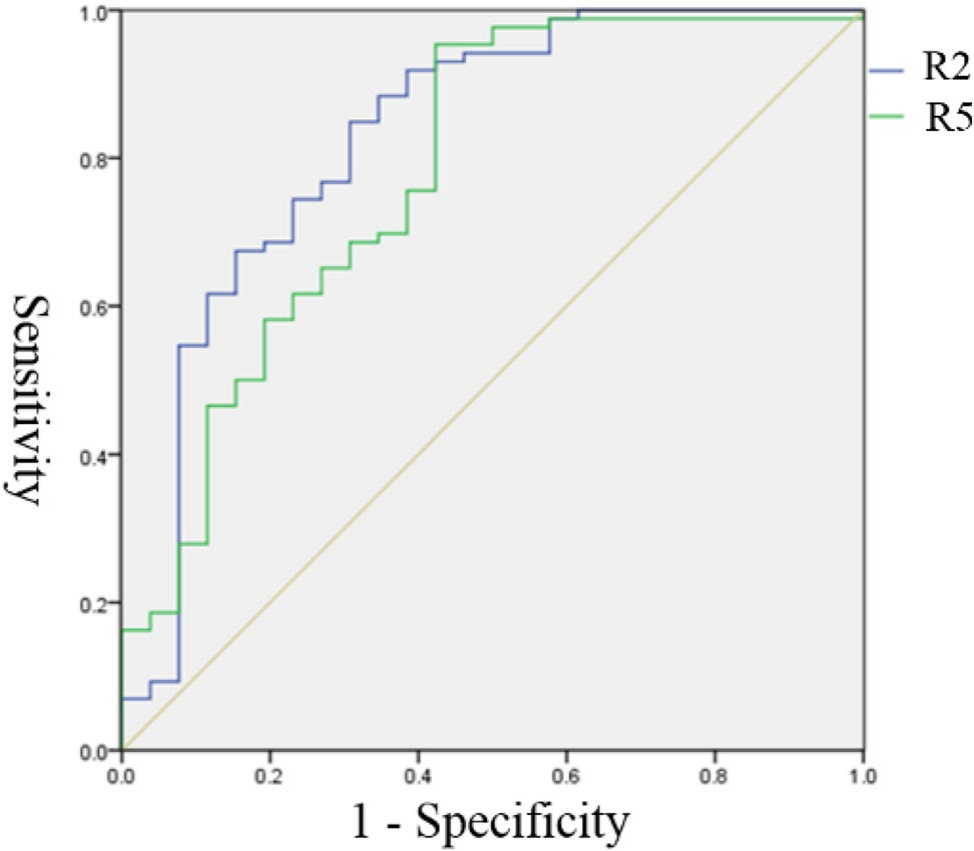
ROC curve analysis of R2 and R5 for the diagnosis of bone healing.

To clarify the diagnostic efficacy of R2 and R5 in determining the degree of healing and the critical diagnostic value, the patients were first divided into the bone healing group (group A) and the nonunion group (groups B and C). The ordinate represents the true positive (sensitivity), and the abscissa represents the false positive (1-specificity). The ROC curve for predicting fracture healing was drawn with different R2 and R5 values as diagnostic cutoff points. The area under the curve of R2 and R5 was 0.858 (95% CI: 0.788–0.929) (*p* < 0.001) and 0.784 (95% CI: 0.700–0.868) (*p* < 0.001), respectively (Fig. 5). R2 was more effective in diagnosing bone healing than R5. The best diagnostic node was then selected according to the maximum Youden index method. The best value of R2 for the diagnosis of bone healing was 0.836 (sensitivity, 0.796; specificity, 0.841), and the maximum Youden index was 0.637. The best value of R5 for the diagnosis of bone healing was 0.796 (sensitivity, 0.653; specificity, 0.175), and the maximum Youden index was 0.478.

**Figure 6:**
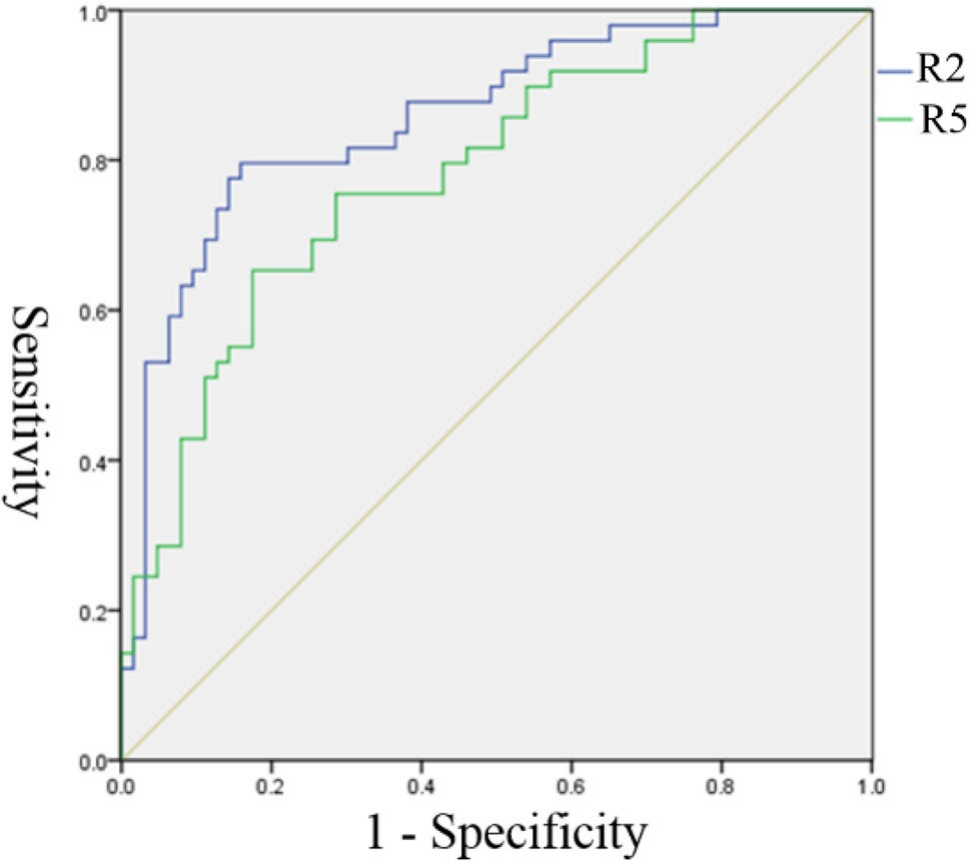
ROC curve analysis of R2 and R5 for the diagnosis of bone nonunion.

We then divided all patients into the nonunion group (group C) and the bone healing group (groups A and B) for ROC curve analysis to determine the diagnostic efficacy of R2 and R5 for nonunion. The area under the curve of R2 and R5 was 0.831 (95% CI: 0.727–0.934) (*p* < 0.001) and 0.781 (95% CI: 0.671–0.892) (*p* < 0.001), respectively (Fig. 6). R2 was more effective in diagnosing bone nonunion than R5. The best diagnostic value of R2 for bone nonunion was 0.743 (sensitivity, 0.849; specificity, 0.308), and the maximum Youden index was 0.541. The best diagnostic value of R5 for bone nonunion was 0.582 (sensitivity, 0.953; specificity, 0.423), and the maximum Youden index was 0.530.

### Typical case reports

*Case 1*: A patient with poor bone healing achieved bone healing after periodic observation.

**Figure 7:**
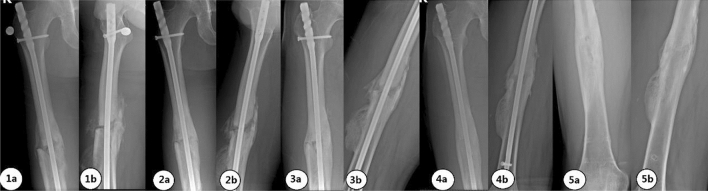
X-ray films at 6 (**1**), 15 (**2**), 23 (**3**), and 35 (**4**) months after fixation and after removal of the internal fixation (**5**). Anterior view (**a**). Lateral view (**b**).

**Figure 8:**
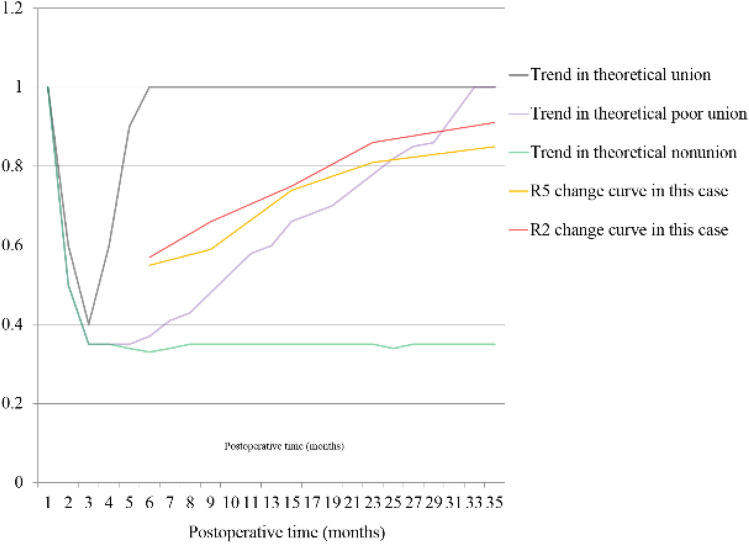
Schematic diagram of R2 and R5 during follow-up in case 1.

A 25-year-old male patient with a right femoral shaft fracture underwent intramedullary nail fixation. X-ray and CT examinations at 6 months after surgery showed poor bone healing. X-ray and CT examinations at 9, 15, and 23 months after surgery all indicated poor bone healing, but the differential ratios R2 and R4 continued to increase (Fig. 8). The imaging examination at 35 months postoperatively indicated bone healing, and the internal fixation was removed. No complications were observed 1 year after the operation. The follow-up imaging data are shown in Fig. 7.

The CT data at each follow-up after the operation were analyzed, and the corresponding R2 values were obtained. As the healing process progressed, R2 gradually increased (Table 2, Fig. 8).

**Table 2:** R2, R4 and R5 data at different time points in case 1.

Postoperative time (months)	6	9	15	23	35
R2	0.57	0.66	0.75	0.86	0.91
R4	0.96	0.89	0.98	0.94	0.93
R5	0.55	0.59	0.74	0.81	0.85

*Case 2*: A patient with poor bone healing continued to be observed, and no growth was observed, but bone healing was achieved after bone graft surgery.

**Figure 9:**
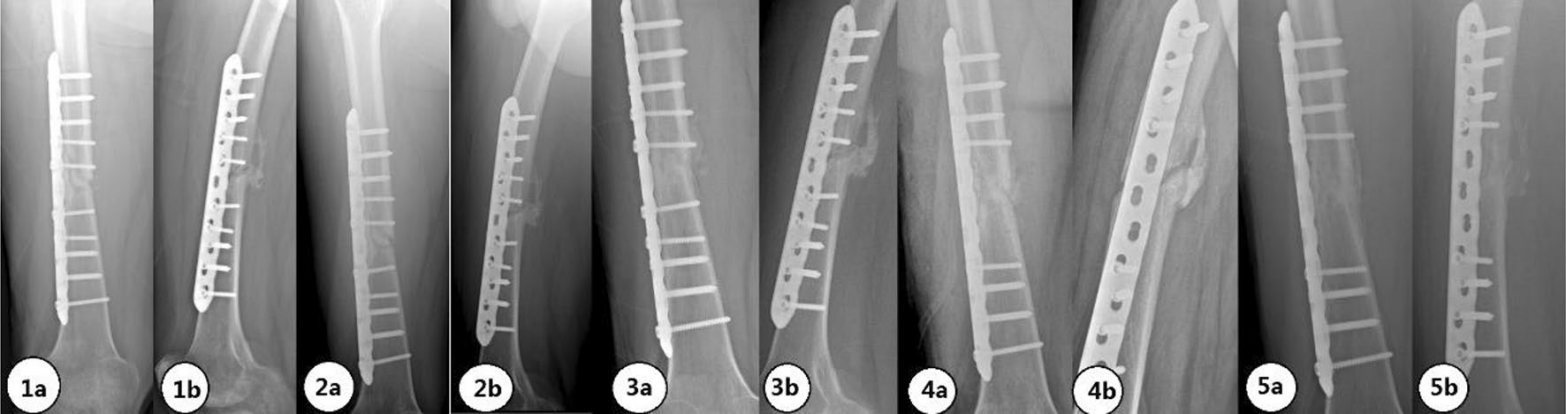
X-ray images at 6 (**1**), 7 (**2**), and 10 (**3**) months after the fixation operation, after bone grafting (**4**), and at 19 months after the fixation surgery (**5**). Anterior view (**a**). Lateral view (**b**).

A 37-year-old male patient with a right femoral shaft fracture underwent open reduction and internal fixation. Imaging examinations at 6, 7, and 10 months (Fig. 9) after surgery all showed that no obvious increasing trend in R2. Combined with the CT findings, bone nonunion was considered. Iliac bone grafting was performed at 10 months after the fixation operation. At 17 months (7 months after bone grafting) and 19 months (9 months after bone grafting) (Fig. 9) after the fixation surgery, bone healing was considered by physical and imaging examinations.

**Figure 10:**
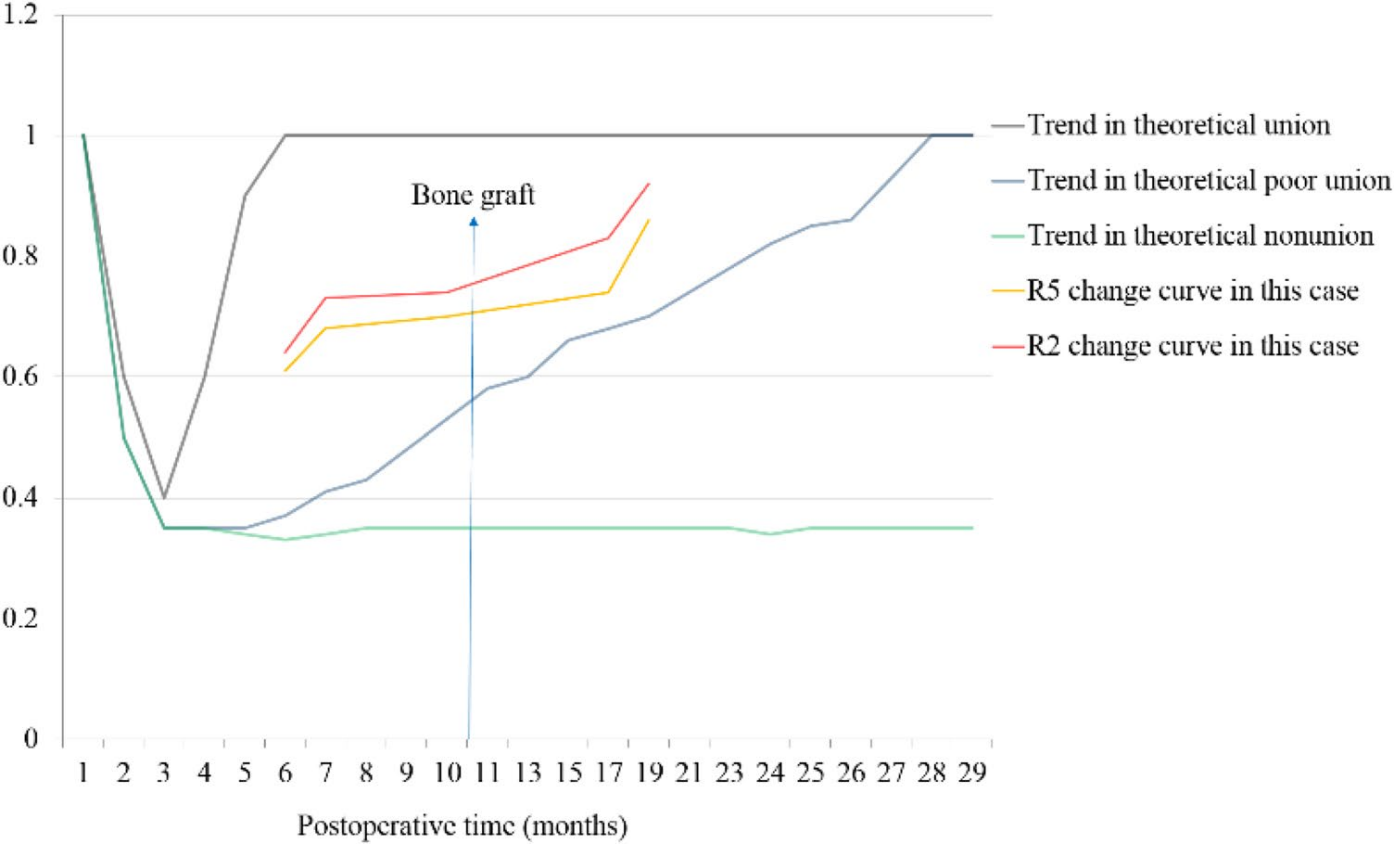
Schematic diagram of R2 and R5 during follow-up in case 2.

R2 still did not reach the healing standard at 7 months after internal fixation, and R2 did not change much during the observation period. However, R2 gradually increased after the bone graft surgery at 10 months after the fixation surgery (Table 3, Fig. 10).

**Table 3:** R2, R4, and R5 at different time points in case 2.

Postoperative time (months)	6	7	10	17	19
R2	0.64	0.73	0.74	0.83	0.92
R4	0.96	0.93	0.95	0.89	0.94
R5	0.61	0.68	0.70	0.74	0.86

## Discussion

The ratio analysis method can visually and quantitatively reflect the process and degree of bone healing based on the bone wall thickness. Fracture healing is a complex regeneration process is similar to bone development except for the initial bleeding and inflammation stage, accompanied by changes in the wall thickness of the bone tissue surrounding the fracture^14^. It is a process of continuous changes in bone density and thickness. At the fracture site, a cartilage callus first forms around the fracture. At the fractured end of the bone shaft, bone can be directly formed through intramembranous ossification^15^, and cartilage in the fracture space can be transformed into woven bone through endochondral ossification. Osteoclasts and osteoblasts work alternately to transform woven bone into lamellar bone^16^. Mineralization of the callus leads to an increase in bone mass and cortical bone thickness. The thickness of bone is closely related to its mechanical properties. Therefore, changes in the bone thickness at the fracture site can reflect the process of bone healing. Existing research shows that there is a linear correlation between bone density and cortical bone thickness^17^. To a certain extent, the thickness of bone can provide information reflecting both the morphology and density of bone. Our research also demonstrates a significantly stronger correlation between R2 and the healing degree than between R5 and the healing degree. Moreover, R2 was more effective in diagnosing bone healing and nonunion than R5. Bone tissue wall thickness changes are closely related to fracture occurrence and bone healing.

Clinically, there are still no uniform standards for judging bone healing^18^. Most standards are qualitative standards, and the judgment process is highly subjective. Clinically, judging whether a patient's fracture is healed usually depends on factors such as physical and imaging examination findings and patients’ subjective feelings. Imaging evaluations mainly rely on X-ray examinations and usually include assessment of the bone callus, trabecular bridge fractures, disappearance of the fracture line, and continuity of the cortical bone. However, recent studies have shown that the diagnosis in terms of the patient's clinical manifestations, imaging findings, and subjective feelings may be inconsistent^19^. To quantitatively judge the degree of bone healing, Whelan proposed a method for scoring the presence of a callus and fracture line on four sides of the tibia in 2010, which is known as the Radiographic Union Score in tibial (RUST) fractures^20^. RUST is widely recognized by clinicians. Later, the Radiographic Union Score for hip (RUSH) fractures^21^ and the Radiographic Union Score for radial (RUSS) fractures^22^ were established for the judgment of hip and radial fracture healing. However, imaging healing scoring systems need to separately determine whether the callus forms and whether the fracture line disappears. These systems are still subjective and yield a semiquantitative judgment index, which has certain limitations. The present work analyzed the median wall thickness at different degrees of healing and identified the cutoffs for bone healing, poor bone healing, and bone nonunion. In our analysis, real-time quantitative CT data were directly without manual intervention. This quantitative and objective analysis reduces the influence of subjective factors on the results.

Some scholars have used finite element analysis to simulate the process, influential factors, and degree of bone fracture healing and determine whether the internal fixation can be removed. Long bone CT data could be used to verify the material properties of each component of the bone. Our previous research applied finite element simulation technology to study the theoretical changes in the load-bearing capacity of the target segment for bone healing research. By the rapid modeling of bone healing, finite element analysis can be used to compare pre- and postoperative models. Additionally, by applying an appropriate static load and constraints, the stress distribution and whether refracture will occur after the internal fixation is removed can be analyzed to guide clinical treatment. Finite element analysis and modeling, mesh generation technology and related calculation methods are becoming increasingly mature^23^. However, the material needs to be assigned, and the organization of the model components needs to be simple for the assignment process. Therefore, models can be partially distorted. Additionally, the calculations can be cumbersome and time consuming, which involve certain drawbacks. In this work, Mimics software was used to personalize the modeling and analysis of the wall thickness of the target bone segment after downloading the patient's CT data. In the process of individualized modeling, we only need to remove artifacts, select the same bone CT value range, and apply a triangular mesh; then, wall thickness analysis can be performed. There is no need for bone material assignment or model optimization, which greatly reduces the model distortion and the analysis complexity, in turn greatly simplifying the analysis and shortening the required time. In addition, models can be examined separately using CT data at different time points to reflect changes in the bone wall thickness over time and indicate whether bone healing is progressing. Therefore, the ratio of the wall thickness and the healing index of the affected side and the healthy side in the reconstruction model of individualized CT data can quantitatively reflect the degree of bone healing in real time.

Bone healing and nonunion can be judged by critical points, and patients with poor healing can be identified by periodic observation over time. In this group of cases, we used the wall thickness analysis technique to compare the proportions of the postoperative recovery of lower limb fractures. Analyzing changes in R2 allowed the differential diagnosis of bone healing, poor bone healing and bone nonunion. Thus, this method provides a clear and objective judgment index and can be used to effectively and intuitively map changes in the wall thickness during bone healing to gauge the progress of the healing process. To reduce the measurement error, we selected the ratio of the median wall thickness on the affected side after the simulated removal of the internal fixation to that on the healthy side obtained under the same scanning and threshold conditions as the evaluation index. The results show that fracture healing can be identified when R2 reaches 0.84, nonunion can be identified when R2 is less than 0.74, and poor healing can be identified when R2 is between 0.74 and 0.84. Patients with poor healing were clinically followed up systematically over time, and changes in R2 were periodically observed. If R2 continues to increase above 0.84, removal of the internal fixation can be considered, and if R2 shows little change, bone grafting can be considered.

This was a retrospective and single-center study, and the number of cases was small. In future prospective studies, we will increase the number of cases. Due to the short research duration and the lack of large-sample verification and in-depth verification by animal experiments, it is necessary to also consider other indicators to make correct clinical judgments. This method is only an auxiliary judgment tool.

## Conclusions

The results of this study showed that individualized differential ratio method can be used to quantitatively evaluate the fracture healing state, with the median wall thickness ratio as a more intuitive and reliable judgment index. For patients who are difficult to judge the fracture healing state through imaging examinations, continuous observation of the change trend of R2 can assist in judging the fracture healing state. Overall, this method provides a reliable reference for the clinical quantitative judgment of bone healing and subsequent treatment options.

## Data Availability

The datasets used and analysed during the current study are available from the corresponding author on reasonable request.
